# Virtual reality in undergraduate and postgraduate nursing education: a scoping review protocol integrating data mining for topic discovery

**DOI:** 10.1016/j.mex.2025.103391

**Published:** 2025-05-24

**Authors:** Silvia Ronchi, Rosario Caruso, Diletta Fabrizi, Michela Luciani, Celeste M. Alfes, Noriyo Colley, Arianna Magon, Gianluca Conte, Anna Valli, Stefano Terzoni, Silvia Cilluffo, Maura Lusignani, Davide Ausili

**Affiliations:** a Department of Medicine and Surgery, University of Milano-Bicocca, Milano, Italy; b Department of Biomedical Sciences for Health, University of Milan, Milan, Italy; c Health Professions Research and Development Unit, IRCCS Policlinico San Donato, San Donato Milanese, Italy; d Case Western Reserve University, Frances Payne Bolton School of Nursing, Cleveland, USA; e Department of Health Sciences, School of Medicine, Hokkaido University, Japan; f Mosaic Factor, Barcelona, Spain

**Keywords:** Virtual reality, Nursing education, Nursing, Learning

## Abstract

Virtual Reality (VR) encompasses a range of computer-based technologies that simulate complex scenarios, offering immersive, experiential learning in a controlled virtual environment. In nursing education, VR has the potential to enhance both technical and non-technical competencies. However, the existing literature on VR in nursing education is fragmented, making it challenging to fully grasp its scope, applications, and emerging trends. This scoping review protocol outlines a systematic approach to mapping the existing literature on the use of VR in undergraduate and postgraduate nursing education. Following Joanna Briggs Institute guidelines and the PRISMA-ScR 2020 framework, the review will include studies published in English, Spanish, or Italian, as well as those with an accessible HTML version to enable accurate automated translation and eligibility assessment. A comprehensive search will be conducted across PubMed, Scopus, CINAHL, and EMBASE, with no time restrictions. Two independent reviewers will assess study eligibility and extract data using a standardized form. Additionally, data mining techniques, including Latent Dirichlet Allocation enhanced by Bayesian optimization, will be employed to identify trends and emerging topics in the field, providing valuable insights for educators, researchers, and policymakers.

• This scoping review protocol outlines the methodology for systematically mapping the existing literature on VR in nursing education, providing a comprehensive overview of its applications at undergraduate and postgraduate levels.
• Advanced data mining techniques will be applied to uncover emerging trends and key topics, enhancing the understanding of VR’s evolving role in nursing education.
• Findings will offer methodological and practical insights, supporting educators, researchers, and policymakers in optimizing and expanding VR-based learning strategies in nursing.

## Graphical abstract

**Figure:**
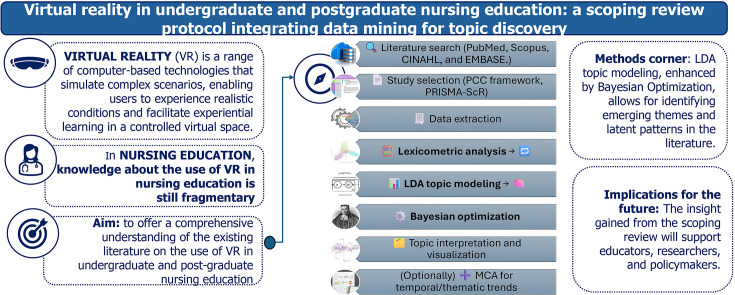

Specifications table

**Table:** 

Subject area:	Medicine and Dentistry
More specific subject area:	Nursing
Name of your protocol:	Mapping virtual reality in nursing education: A scoping review protocol with data mining for trends and topic discovery
Reagents/tools:	None.
Experimental design:	Not applicable.
Trial registration:	Not applicable.
Ethics:	This scoping review does not involve human participants, animal experiments, or data collected from social media platforms. It will utilize data from previously published studies. Therefore, ethical approval and informed consent are not required.
Value of the Protocol:	• This protocol outlines methodological steps to comprehensively synthesize the existing literature on virtual reality in undergraduate and postgraduate nursing education, mapping key applications, benefits, challenges, and emerging topics. • With the integration of Latent Dirichlet Allocation (LDA) topic modelling with Bayesian Optimization, this review will identify hidden patterns and trends, offering a more nuanced understanding of the evolving role of VR in nursing education. • The findings will contribute both methodologically and practically, equipping educators, researchers, and policymakers with data-driven insights to enhance and expand VR-based learning strategies in nursing.

## Background

Virtual reality (VR) encompasses a wide variety of computer-based technologies and applications used to create an interactive world with immersive, highly visual, 3D characteristics that allow the participant to look around and navigate within a seemingly real or physical world [[1], [2], [3]]. This immersive environment is used to simulate complex scenarios, enabling users to experience realistic conditions that are otherwise difficult to replicate in traditional training settings [4]. Furthermore, VR fosters experiential learning by engaging users in dynamic, hands-on interactions that enhance retention and skill acquisition in a controlled, safe virtual space [5].

The technological advances of the last few decades have made it possible to create learning opportunities based on software and digital platforms [6]. These advancements became particularly significant during the COVID-19 pandemic, as social distancing measures and the disruption of in-person clinical learning activities necessitated alternative strategies. VR emerged as a crucial tool not only to provide healthcare services that could not be delivered through traditional methods [2] but also to address the challenges faced in education during this period. VR enabled educators to maintain the continuity of learning by offering remote and flexible training solutions, allowing students to access immersive simulations without needing physical presence [7].

In nursing education, the pandemic accelerated the adoption of VR to mitigate the negative effects of limited access to traditional training methods, such as mannequin-based simulations or clinical internships. Educators utilized VR to ensure that students could continue to develop their skills and competencies safely despite the constraints imposed by the pandemic. This technology provided opportunities for independent learning and supported the acquisition of critical skills, helping students maintain preparedness for professional practice [2,8]. In addition, while VR is commonly used to teach technical skills, it also holds the potential for developing non-technical or “soft skills,” such as communication, decision-making, and ethical reflection [9,10].

Despite numerous systematic reviews addressing the efficacy of VR on various outcomes [5,4,[11], [12], [13], [14], [15], [16]], the abundance of both primary and secondary studies has led to a fragmented body of literature. Notably, the recent scoping review by Vogelsang et al. [16] focused specifically on immersive virtual reality in undergraduate nursing education, offering valuable thematic insights into user experience and educational outcomes. However, its scope was limited to immersive modalities, defined by the use of head-mounted displays and haptic technology: it excluded non-immersive VR applications, postgraduate learners, and broader conceptualizations of virtual simulation.

In this landscape, adopting a broader perspective by including both immersive and non-immersive VR, as well as undergraduate and postgraduate contexts, combined with data mining techniques, could offer an opportunity to clarify the existing knowledge and synthesize insights [17]. To date, no scoping reviews in this field have employed such analytical approaches, underscoring the novelty and added value of the present study. A preliminary search conducted in February 2025 across PubMed, Scopus, Web of Science, and CINAHL confirmed the absence of scoping reviews with this dual focus, which encompasses a comprehensive scope of the review regarding VR in undergraduate and postgraduate nursing education, coupled with automated topic modeling. This gap supports the originality and necessity of the proposed review. Therefore, this protocol outlines the methods used to conduct a scoping review to map the current literature on VR in nursing education and apply data mining techniques to identify emerging topics.

## Description of protocol

### Design and research questions

The scoping review will be conducted according to the Joanna Briggs Institute guidelines [18], using the Preferred Reporting Items for Systematic Reviews and Meta-Analyses extension for Scoping Reviews (PRISMA-ScR) 2020 framework to guide the literature selection and review process [[19], [20], [21], [22]].

The main research question guiding the scoping review was constructed based on the elements of the Population, Concept, Context (PCC) framework, which will later define the eligibility criteria for the literature [20,21]. The main research question is: “What is the current state of literature on the use of virtual reality in undergraduate and postgraduate nursing education?”

As is typical in scoping reviews, the main question is intentionally broad to comprehensively map the available evidence. To provide greater specificity and structure to the investigation, secondary questions are necessary to explore key aspects of the topic in more detail, such as geographic and organizational contexts, types of literature, outcomes, and emerging themes. Several secondary research questions were then formulated:

- RQ1: In which geographic contexts has virtual reality been used in nursing education?
- RQ2: In what clinical and organizational contexts has virtual reality been developed and used in nursing education?
- RQ3: What type of literature has been produced?
- RQ4: For which types of outcomes has virtual reality been used?
- RQ5: What is the overlapping rate of primary studies included in the subset of included secondary literature focused on the efficacy of VR (a subgroup of the available metanalyses)?
- RQ6: What are the advantages and disadvantages of this type of training?
- RQ7: What kind of technology is needed for this kind of training?
- RQ8: What are the emerging topics in the literature identified using data mining techniques?
- RQ9: Is there a relationship between themes and time of publication? Are there emerging themes depending on the publication period?

While all the research questions stated above align broadly with the exploratory purpose of a scoping review (i.e., mapping the literature on VR applications in nursing education), we acknowledge that certain sub-questions (specifically RQ5, RQ8, and RQ9) introduce analytical elements typically associated with other review methodologies. In particular, RQ5 utilizes an approach consistent with an umbrella review by investigating overlaps among primary studies cited in existing systematic reviews [23]. This approach helps to reveal potential redundancies and gaps within secondary literature, enriching the interpretative context of the scoping review findings. Similarly, RQ8 and RQ9 require targeted analytical methods, leveraging advanced data-driven techniques such as text mining and topic modeling, to uncover latent thematic structures and temporal dynamics within the literature [24].

These analytical extensions, however, do not compromise the methodological coherence or exploratory intent of the scoping review framework. Rather, the inherent flexibility of scoping reviews, as recommended by the JBI guidelines, makes this methodology particularly appropriate to incorporate and synthesize diverse types of evidence and analytical approaches [24]. Compared to systematic or umbrella reviews, which are typically narrower and oriented towards confirming effectiveness or aggregating existing quantitative syntheses, a scoping review provides a broader and more inclusive mapping, essential given the heterogeneity and evolving nature of the field of VR in nursing education. Thus, these complementary analytical methods are explicitly integrated to enrich and deepen the mapping process, rather than to substitute for more specialized review types, as employed in a recent published scoping review [24]. This methodological integration clearly delineates the objectives and boundaries of each sub-question, maintaining the overall rigor and coherence of the scoping review approach.

### Eligibility criteria

In accordance with the Joanna Briggs Institute criteria, eligibility criteria were defined based on the PCC framework. This scoping review will consider all studies conducted within nursing education settings without imposing geographic or educational level restrictions. This includes undergraduate nursing students and postgraduate registered nurses, ensuring the review captures a comprehensive range of educational contexts (population). The concept underpinning this review is virtual reality simulation, focusing on mapping the existing literature that explores the application of VR in nursing education at both undergraduate and postgraduate levels. To facilitate a thorough and inclusive analysis, the review will include studies from nursing education programs worldwide, without limitations on territorial or thematic scope or time (to detect time-sensitive changes in literature). This approach allows for identifying potential variations across different geographical and institutional settings, contributing to a holistic understanding of how VR is utilized in nursing education globally.

All publications responding to the PCC framework will be considered for inclusion, regardless of the methodological approach used. Empirical studies (quantitative, qualitative, or mixed methods) will be included, as well as literature reviews, theoretical papers, commentaries, editorials, conference proceedings, dissertations, and policy documents. All publications on virtual reality use in nursing focused on patient education will be excluded.

There were no limitations posed on the time frame or language of the publications considered in this review. However, the exclusion criteria are singularly focused on accessibility constraints: studies not available in English, Italian, or Spanish or lacking an HTML version will be excluded. The absence of an HTML version limits the feasibility of translating non-English texts, which is essential for accurately evaluating the content’s relevance and alignment with the objectives of the scoping review.

### Definition and scope of VR technologies

In the context of this review, VR is understood as a broad spectrum of computer-generated environments that enable user interaction for the purpose of experiential learning in nursing education [5,4,[11], [12], [13], [14], [15], [16]]. This inclusive conceptualization allows the review to capture the full range of technologies currently adopted or explored within educational contexts. The scope includes both immersive and non-immersive systems. Immersive VR is characterized by the use of head-mounted displays, with or without additional sensory modalities such as haptic feedback or spatial audio, designed to provide a high level of user presence within a simulated environment. Non-immersive VR refers to monitor-based systems, including desktop virtual environments and interactive 3D simulations that allow user navigation through traditional input devices. The review also considers advanced projection systems, such as Cave Automatic Virtual Environment (CAVE) environments and simulation stations, provided they engage users in real-time interaction within a digitally constructed three-dimensional space. Studies that focus exclusively on investigating device performance without an educational component relevant to nursing are considered outside the scope of this review.

### Type of sources

This scoping review will include all studies identified in the literature search that meet the inclusion and exclusion criteria without restrictions on the methodological approach used. If the comprehensive search yields a limited number of eligible records, grey literature will be reviewed to identify hidden or unpublished sources.

### Search strategy

Potentially relevant records will be identified by searching the following electronic databases: PubMed, Scopus, CINAHL, and EMBASE. The search strategy will combine controlled vocabulary terms and keywords with specific search terms, synonyms, and Boolean operators to combine the concepts effectively. The search strings, originally generated for PubMed and later replicated in other databases, were created with the goal of maximizing sensitivity and specificity to obtain the greatest number of relevant records. The complete preliminary search strategy for all databases is provided in Table 1. The databases will be re-searched after the release of the protocol to include any newly published records. Additionally, a supplementary search will be conducted on Google Scholar to identify any potentially relevant studies. A manual search of the references cited in the included records will also be performed to ensure comprehensive coverage of the literature.

**Table 1:** Preliminary search strategy in biomedical databases.

Database	Preliminary query	Records Feb 28th; 2025
PubMed	("Virtual Reality"[Mesh] OR ("virtual realit*"[Title/Abstract] OR "Virtual Reality"[Majr])) AND (("Education"[Mesh] OR "education"[tiab] OR ‘learning’[tiab] OR ‘instruction’[tiab] OR ‘problem solving’[tiab]) AND ("Nurses"[Mesh] OR "Nursing"[tiab] OR ‘nurse* student*’[tiab] OR ‘student* nurse*’[tiab] OR "undergraduate nurs*"[tiab] OR "pre-licensure nurs*"[tiab]))	478 no filters or time limits
CINAHL	((MH "Virtual Reality+") OR ((TI "virtual realit*" OR AB "virtual realit*") OR (MM "Virtual Reality+"))) AND (((MH Education+) OR (TI education OR AB education) OR (TI ‘learning’ OR AB ‘learning’) OR (TI ‘instruction’ OR AB ‘instruction’) OR (TI” ‘problem solving’” OR AB” ‘problem solving’”)) AND ((MH Nurses+) OR (TI Nursing OR AB Nursing) OR (TI” ‘nurse* student*’” OR AB” ‘nurse* student*’”) OR (TI” ‘student* nurse*’” OR AB” ‘student* nurse*’”) OR (TI “undergraduate nurs*” OR AB “undergraduate nurs*”) OR (TI “pre-licensure nurs*” OR AB “pre-licensure nurs*”)))	796 no filters or time limits
Embase	(‘virtual reality'/exp OR ‘virtual realit*':ti,ab OR ‘virtual reality'/exp/mj) AND (‘education'/exp OR education:ti,ab OR ‘learning':ti,ab OR ‘instruction':ti,ab OR ‘problem solving':ti,ab) AND (‘nurses'/exp OR nursing:ti,ab OR ‘nurse* student*':ti,ab OR ‘student* nurse*':ti,ab OR ‘undergraduate nurs*':ti,ab OR ‘pre-licensure nurs*':ti,ab)	565 no filters or time limits
Scopus	(INDEXTERMS ("Virtual Reality") OR (TITLE-ABS ("virtual realit*") OR INDEXTERMS ("Virtual Reality"))) AND ((INDEXTERMS (education) OR TITLE-ABS (education) OR TITLE-ABS (‘learning') OR TITLE-ABS (‘instruction') OR TITLE-ABS ("‘problem solving'")) AND (INDEXTERMS (nurses) OR TITLE-ABS (nursing) OR TITLE-ABS ("‘nurse* student*'") OR TITLE-ABS ("‘student* nurse*'") OR TITLE-ABS ("undergraduate nurs*") OR TITLE-ABS ("pre-licensure nurs*")))	864 no filters or time limits

The search strategies reported in Table 1 represent preliminary queries intended to provide a comprehensive initial coverage of the literature. Adjustments and refinements will be implemented before conducting the final literature searches. In particular, to enhance the comprehensiveness and relevance of retrieved records, especially given the focus on educational interventions and skill acquisition, the keyword “training”[tw] and related synonyms will be added to the final search strings. Additional adjustments may be considered following initial screening results and consultation with subject matter experts, ensuring maximum sensitivity and specificity in capturing relevant studies.

All search results will be systematically imported into reference management software, such as Zotero and Rayyan, to ensure efficient organization and handling of records. Duplicate entries will be identified and removed before the screening process. Following this, two independent reviewers will screen the records based on their titles and abstracts to determine initial eligibility in accordance with the predefined inclusion and exclusion criteria. Any discrepancies during this phase will be resolved through discussion or consultation with a third reviewer, if necessary, to ensure consistency and reliability in the selection process.

In the subsequent stage, full-text screening will be conducted independently by the same two reviewers to assess the eligibility of each study in greater detail. Publications that do not meet the inclusion criteria will be excluded, and the reasons for exclusion will be documented systematically to provide transparency. This ensures that all decisions regarding inclusion or exclusion are well-justified and reproducible.

To ensure the rigor and transparency of the selection process, the entire workflow will be summarized in a flowchart in accordance with the PRISMA 2020 guidelines. This flowchart will detail the number of records identified, duplicates removed, titles and abstracts screened, full-text articles assessed for eligibility, and studies ultimately included in the review [19].

### Data extraction

Data extraction will be performed independently by two authors. The authors will evaluate each publication and proceed with data extraction, ensuring methodological rigor and reliability in the data collection process. A consensus discussion will be used to merge and compare the extractions and views of the reviewers involved. Any disagreements between the reviewers will be resolved through discussion or consultation with an additional reviewer, if necessary.

Data extraction will be conducted using tools specifically developed for this scoping review. The tools will consist of a set of data extraction tables, created using Microsoft Excel, that includes information about the participants, the purpose, conceptual focus, and context of the study, the methodology, and the main findings related to the research question. A preliminary version of the instruments designed for data extraction is presented in Table 2, Table 3. Any subsequent changes will be explained in the scoping review documentation. In addition, the data extraction tool presented in Table 2 is designed to facilitate the creation of a textual corpus derived from the key findings of each study. This corpus will serve as a dataset for applying advanced analytical techniques based on a Bayesian optimization approach to identify themes within the textual data [25].

**Table 2:** Data extraction instrument.

First author, title and year	Study design/Knowledge synthesis method	Aim	Research focus	Population/sample characteristics	Context (geographical area, clinical setting or theme/scenario)	Concept (technologies used)	Outcomes	Pros and Cons	Key Findings
[article n]	[concise extraction]	[concise extraction]	[concise extraction]	[concise extraction]	[concise extraction]	[concise extraction]	[no limits to the extracted words to produce a textual corpus]	[concise extraction]	[no limits to the extracted words to produce a textual corpus]

**Table 3:** Data extraction instrument: primary studies included in secondary literature.

Review 1	
	Study 1
	Study 2
	Study n

Review 2	
	Study 1
	Study 2
	Study n

Review n	
	Study 1
	Study 2
	Study n

### Data analysis

Starting from the data extracted in Table 2, Table 3, a narrative analysis will be performed to answer primary and secondary research questions. Regarding RQ1 and RQ2, the data extracted in the “Context” column of Table 2, indicating the geographic area, the clinical setting, and the scenarios developed, will be analyzed. To respond to RQ3, the narrative analysis will focus on the data contained in the column entitled “Study design/method of knowledge synthesis”. Regarding RQ4, the analysis will focus on the data of interest included in the “Outcomes” column. To emphasize the primary advantages and disadvantages, as well as the most frequently employed technologies in the implementation of virtual reality (RQ6 and RQ7), the data presented in the “Concept” and “Pros and Cons” columns will be examined through a narrative lens. The contents of Table 3 are analyzed to provide an answer to RQ5, calculating the overlapping rate of primary studies included in the subset of secondary literature included.

To respond to RQ8 and RQ9, the analytical process begins with a preliminary lexicometric analysis aimed at gaining a foundational understanding of the textual data extracted from the included studies. This involves tokenizing the textual corpus, cleaning the data by removing stopwords, punctuation, numbers, and whitespace, and stemming words to their root forms. Key metrics from this analysis include the number of texts, total word occurrences (N), number of unique word forms (V), and hapax legomena (words that occur only once). Additional measures, such as the mean occurrences per text and the type-to-token ratio (V/N), provide insights into the richness and variability of the textual data. This lexicometric analysis provides an essential overview of the corpus, offering insights into its structure and complexity, which informs the subsequent stages of topic modeling [26].

After performing the preliminary lexicometric analysis, Latent Dirichlet Allocation (LDA) is applied to identify the main topics embedded within the textual corpus [27]. LDA is a probabilistic topic modeling technique that analyzes patterns of word co-occurrence across the dataset to uncover latent themes. LDA provides a structured representation of the textual data, enabling the identification of meaningful patterns by assigning words to topics and topics to documents.

The first step in this process involves data preparation. The cleaned textual corpus is transformed into a Document-Term Matrix (DTM), which serves as the input for the LDA model. The DTM is a sparse matrix where each row corresponds to a document, and each column represents the frequency of a specific term across the corpus. This transformation ensures that the data is in a suitable format for computational analysis.

A critical aspect of LDA is determining the optimal number of topics, as this significantly impacts the interpretability and coherence of the results. Multiple configurations are tested using the “ldatuning” library to identify the best-fitting model. This process involves evaluating the model’s performance across several metrics designed to assess different aspects of topic modeling quality:

(a) Griffiths2004: This metric evaluates the likelihood of the model by measuring how well the data fits the specified number of topics. Higher likelihood values indicate a better match between the model and the dataset, suggesting that the chosen number of topics provides a more accurate representation of the data [28].
(b) CaoJuan2009: This metric focuses on topic coherence, assessing the semantic similarity between the most probable words within each topic. Coherence is critical for ensuring that the topics are meaningful and interpretable. Lower values of this metric indicate higher coherence and better topic quality [29].
(c) Arun2010: This metric evaluates the divergence between document-topic and topic-word distributions. It assesses whether the topics are sufficiently distinct from one another. Smaller divergence values suggest better separation and uniqueness among topics [30].
(d) Deveaud2014: This metric considers the overall quality of the model based on entropy, which measures the degree of randomness in the topic distribution. Lower entropy values typically indicate a more stable and interpretable model [31].

These metrics collectively guide the selection of the optimal number of topics, balancing the trade-off between maximizing interpretability and ensuring sufficient granularity in the results. The rationale for selecting these metrics is to comprehensively evaluate topic modeling quality from multiple complementary perspectives: likelihood (fit), coherence (interpretability), distinctiveness (topic uniqueness), and stability (consistency of topic distributions). Given that each metric focuses on distinct aspects of model quality, their collective interpretation provides a balanced, multi-dimensional performance assessment [24]. Practically, the final decision on the optimal number of topics will involve examining these metrics collectively rather than individually and qualitatively assessing the interpretability and practical relevance of the resulting topics. This qualitative step ensures that chosen topics are not only statistically optimal but also conceptually meaningful and relevant to the aims of the scoping review. Such a comprehensive approach mitigates the risk of over-reliance on any single metric and promotes robust and interpretable results.

Once the number of topics is determined, Bayesian Optimization is employed to fine-tune the LDA model’s hyperparameters, including the topic-word and document-topic distributions. Bayesian Optimization uses a probabilistic approach to iteratively test combinations of hyperparameters, focusing on configurations that are likely to improve the model’s performance. This process refines the LDA model, ensuring that the resulting topics are not only coherent but also tailored to the dataset’s unique characteristics.

The final step involves visualizing and interpreting the topics. Tools such as word clouds and topic-term matrices are used to display the most relevant terms associated with each topic, providing an intuitive understanding of the themes. These visualizations help assess the coherence and relevance of the topics, ensuring alignment with the research questions. Qualitative evaluation is also conducted to validate that the identified topics reflect meaningful and significant patterns within the literature.

As an additional layer of analysis, Multiple Correspondence Analysis (MCA) may be employed to explore the relationship between the emerging topics and key variables such as “type of publication,” “year of publication,” and other metadata from the studies [32]. MCA enables the representation of complex relationships in a two-dimensional or three-dimensional space by extracting the principal components from the data. This approach provides a graphical interpretation of the associations between topics and variables, revealing patterns that might not be immediately apparent. For instance, MCA helps to illustrate whether certain topics are predominantly associated with specific publication years or types of research outputs, offering deeper insights into trends and patterns within the literature.

### Quality assessment

Although quality assessment is not mandatory in scoping reviews, the authors will evaluate the feasibility of performing a quality assessment of the included records. If deemed appropriate based on the characteristics and quantity of included studies, methodological quality will be assessed using validated, design-specific tools. For quantitative studies, we will consider using the Joanna Briggs Institute Critical Appraisal Checklists for randomized controlled trials, cohort studies, and cross-sectional studies [[33], [34], [35]]. For qualitative studies, we will utilize the JBI Checklist for Qualitative Research or the Critical Appraisal Skills Programme (CASP) qualitative checklist [36,37]. Mixed-method studies, if included, will be assessed using the Mixed Methods Appraisal Tool (MMAT) [38]. This explicit approach aims to provide transparency, ensure methodological rigor, and enhance reproducibility. It will also offer additional insights into the strengths and limitations of the available evidence, supporting a more nuanced interpretation of the scoping review findings.

### Novelty of this study

This scoping review represents a novel approach to synthesizing the literature on the use of VR in nursing education by integrating advanced analytical methods that go beyond traditional descriptive literature reviews. Unlike previous studies, this review will apply LDA topic modeling, enhanced by Bayesian Optimization, to identify emerging themes and latent patterns in the literature. This innovative application of probabilistic modeling allows for a more nuanced understanding of the key topics and trends within the rapidly growing body of research.

Moreover, the review incorporates MCA to explore relationships between identified topics and study variables, such as publication type, geographic context, and year of publication. This combination of advanced techniques provides an unprecedented level of granularity in analyzing the connections between themes and contextual factors. Another unique aspect of this study is the integration of a preliminary lexicometric analysis, which offers foundational insights into the structure and complexity of the textual data before topic modeling. Together, these methods enable the review to uncover hidden patterns and trends, bridging gaps in the fragmented literature on VR in nursing education.

Finally, this is the first study to explicitly employ data mining techniques to map and synthesize the diverse and heterogeneous body of research in this field. The review aims to provide a comprehensive overview and establish a methodological framework that could be adopted for future scoping reviews in health education and beyond by leveraging innovative approaches in framing the knowledge regarding the identified review question. This novel approach ensures that the study contributes both methodologically and substantively to the field, supporting educators, researchers, and policymakers in advancing VR applications in nursing education.

## Protocol validation

The protocol for this scoping review will undergo a rigorous validation process to ensure methodological transparency, reliability, and alignment with best practices in scoping review methodology. This includes adherence to the JBI guidelines and the PRISMA-ScR framework [19,20,22].

To enhance validity, the protocol will be reviewed by a panel of experts in scoping review methodology, virtual reality in education, and nursing research. Their feedback will be incorporated to refine the research questions, eligibility criteria, search strategy, and data analysis methods.

A pilot phase will be conducted to test the study selection and data extraction processes. This involves a preliminary screening of a subset of studies to assess the clarity and consistency of the inclusion and exclusion criteria, as well as the reliability of the data extraction tool. Any issues identified during this phase will be addressed by refining the protocol before full implementation.

## Limitations

This scoping review has several limitations. Firstly, while no time restrictions were applied, excluding studies published in languages other than Italian, English, or Spanish, as well as those lacking an accessible full-text HTML version, may result in the omission of relevant research. This constraint potentially limits the generalizability of findings to contexts with different cultural and linguistic backgrounds. Secondly, the reliance on electronic databases as primary sources may exclude studies published in lesser-known journals or grey literature that are not indexed in these platforms, even though efforts will be made to mitigate this through manual searches of references and supplementary searches on platforms like Google Scholar. Lastly, while scoping reviews aim to map the breadth of available evidence rather than appraise its quality, the absence of a systematic quality assessment for all included studies may impact the ability to assess the methodological rigor of the literature comprehensively. Despite these limitations, the review is designed to provide a robust and comprehensive overview of the current state of the literature on virtual reality in nursing education.

## Supplementary material and/or additional information [OPTIONAL]

None

## Declaration of competing interest

The authors declare that they have no known competing financial interests or personal relationships that could have appeared to influence the work reported in this paper.

## CRediT authorship contribution statement

**Silvia Ronchi:** Conceptualization, Methodology, Writing – original draft, Supervision. **Rosario Caruso:** Conceptualization, Methodology, Writing – review & editing, Supervision. **Diletta Fabrizi:** Conceptualization, Methodology, Writing – review & editing, Supervision. **Michela Luciani:** Conceptualization, Methodology, Writing – review & editing, Supervision. **Celeste M. Alfes:** Conceptualization, Methodology, Writing – review & editing, Supervision. **Noriyo Colley:** Conceptualization, Methodology, Writing – review & editing, Supervision. **Arianna Magon:** Conceptualization, Methodology, Writing – review & editing, Supervision. **Gianluca Conte:** Conceptualization, Methodology, Writing – review & editing, Supervision. **Anna Valli:** Conceptualization, Methodology, Writing – review & editing, Supervision. **Stefano Terzoni:** Conceptualization, Methodology, Writing – review & editing, Supervision. **Silvia Cilluffo:** Conceptualization, Methodology, Writing – review & editing, Supervision. **Maura Lusignani:** Conceptualization, Methodology, Writing – review & editing, Supervision. **Davide Ausili:** Conceptualization, Methodology, Writing – review & editing, Supervision.

## Data Availability

No data was used for the research described in the article.
